# Effect of Core-Shell Morphology on the Mechanical Properties and Crystallization Behavior of HDPE/HDPE-*g*-MA/PA6 Ternary Blends

**DOI:** 10.3390/polym10091040

**Published:** 2018-09-19

**Authors:** Lien Zhu, Haoming Wang, Meihua Liu, Zheng Jin, Kai Zhao

**Affiliations:** 1 Key Laboratory of Chemical Engineering Process and Technology for High-efficiency Conversion, College of Heilongjiang Province, College of Chemistry Engineering and Materials, Heilongjiang University, Harbin 150080, China; zle901111@163.com (L.Z.); 18246485755@163.com (H.W.); liumeihua@hlju.edu.cn (M.L.); 2 Laboratory of Microbiology, School of Biological Science and Technology, Heilongjiang University, Harbin 150080, China

**Keywords:** HDPE/HDPE-*g*-MA/PA6 ternary blends, core-shell morphology, toughening mechanism, mechanical properties, crystallization behavior

## Abstract

In this paper, the high-density polyethylene/maleic anhydride grafted high-density polyethylene/polyamide 6 (HDPE/HDPE-*g*-MA/PA6) ternary blends were prepared by blend melting. The binary dispersed phase (HDPE-*g*-MA/PA6) is of a core-shell structure, which is confirmed by the SEM observation and theoretical calculation. The crystallization behavior and mechanical properties of PA6, HDPE-*g*-MA, HDPE, and their blends were investigated. The crystallization process, crystallization temperature, melting temperature, and crystallinity were studied by differential scanning calorimetry (DSC) testing. The results show that PA6 and HDPE-*g*-MA interact with each other during crystallizing, and their crystallization behaviors are different when the composition is different. At the same time, the addition of core-shell particles (HDPE-*g*-MA/PA6) can affect the crystallization behavior of the HDPE matrix. With the addition of the core-shell particles, the comprehensive mechanical properties of HDPE were enhanced, including tensile strength, elastic modulus, and the impact strength. Combined with previous studies, the toughening mechanism of core-shell structure is discussed in detail. The mechanism of the core-shell structure toughening is not only one, but the result of a variety of mechanisms together.

## 1. Introduction

Polymer melt blending with fillers is an effective, widely used way to modify a polymer with the advantage of low cost and simple process, compared to the development of a new polymer or block copolymer [1,2,3,4,5,6]. As the casual characteristics of polymer blending modification, there are many examples of polymer blending modification in the current paper. In summary, most of the blending modification cases improved the tensile strength, toughness, and elastic modulus of the composites. However, not all of the performances can be improved. In other words, increasing one of these performances, often reduced the performance of another.

In order to improve the tensile strength of the material, blending with the fiber is an effective way, due to the stress transfer from polymer matrix to the fibers [7,8,9]. However, the stress transfer is strongly related to the interfacial strength between the fiber and the matrix [10,11]. At present, most of the interfacial strength is not satisfactory, even if adding the coupling agent. Defects caused by the addition of fibers tend to result in a decrease in impact strength [12]. In order to improve the toughness of the polymer material, adding elastomers to the polymer matrix has been reported by many researchers [13,14,15,16,17,18]. The principle is that the elastomers induced the polymer matrix to produce crazing or shear bands to achieve the purpose of fracture energy consumption and ending the propagation of cracks [19,20,21,22,23]. However, this modification tends to result in a decrease in the elasticity modulus and tensile strength of the polymer blends. Some researchers have added rigid particles to the polymer matrix to achieve the purpose of increasing toughness [24,25]. The principle is that the polymer matrix compresses the rigid particles upon stress resulting in larger matrix deformation around the rigid particles or the rigid particles stripping from the polymer matrix, which achieve the purpose of fracture energy absorption [26]. However, the addition of rigid particles will increase the viscosity and density of the system [27,28,29,30].

The core-shell structure in recent years has been widely used to modify polymeric material. Many researchers have predicted the core-shell structure of ternary blends and used it as a toughening agent [31,32]. Compared to the elastomer toughening, it will not reduce the strength and rigidity of the material, but inevitably produce a small amount of loss. The toughening mechanism of the core-shell structure has also been studied, but none of them is particularly comprehensive [33,34,35]. Valera et al. [36] proposed Polymethylmethacrylate/Polypropylene/polystyrene (PMMA/PP/PS) ternary system, where PP/PS core-shell particles disperse in the PMMA matrix. However, their interface strength cannot be guaranteed due to the different surface energy of the components and the absence of covalent bonds between the components. Shen et al. [33] proposed PA6/EPDM-*g*-MA (Maleic anhydride grafted ethylene propylene diene monomer)/HDPE ternary system, where EPDM-*g*-MA/HDPE core-shell particles disperse in PA6 matrix. The interface strength between the shell and matrix indeed increase due to the covalent bond between matrix (PA6) and shell (EPDM-*g*-MA), but the strength and modulus of the EPDM-*g*-MA/HDPE core-shell particles as the dispersed phase are smaller than the matrix, resulting in a decrease in the elasticity modulus and tensile strength of the matrix. In our previous study [31], the PBT (polybutylene terephthalate)/PA6 core-shell particles were chosen as the dispersed phase toughening HDPE, and the dispersed phase have covalent bonds between the core and shell, and the strength and modulus of the dispersed phase are both larger than that of the matrix. However, it just reinforced the interface strength between core (PA6) and shell (PBT), and the interface strength between shell and matrix was still not ideal.

Crystallization behavior is an important property of a polymer, which has an important effect on the properties of the polymer material. In the process of polymer blend melting, it is possible that the crystallization behavior of the individual components is influenced by the interaction among the components. Although the core-shell structure is widely used in polymer modification, few researchers have explored the crystallization behavior of the core-shell structure modified polymer blends detailly. The crystallization behavior of binary blends similar to the HDPE-*g*-MA/PA6 binary blends which act as the core-shell particles in this work have been studied by many researchers [37,38]. However, they usually study a fixed composition ratio. In fact, their crystallization behavior is different when the composition ratio is different.

High-density polyethylene (HDPE), as a semi-crystalline polyolefin, is one of the most common plastics with a wide range of applications [39,40]. However, its applications were limited due to its low mechanical property. In this work, the HDPE-*g*-MA (maleic anhydride grafted high density polyethylene)/PA6 core-shell particles was used to modify the HDPE. The morphology, crystallization behavior, and mechanical properties of the modified HDPE were investigated.

## 2. Materials and Methods

### 2.1. Materials

High density polyethylene (product brand-5000 s) with mass density 0.980 g/cm^3^ was from Daqing Petrochemical Company (Daqing, China). Polyamide 6 (product brand-6mv13) with a mass density of 1.13 g/cm^3^ was obtained from A. Schulman Engineering Plastics Co., Ltd. (Dongguan, China). Maleic anhydride grafted high-density polyethylene (HDPE-*g*-MA), with a grafting degree of 1%, was purchased from Guangzhou Qiansan Chemical Technology Co., Ltd. (Guangzhou, China).

### 2.2. Sample Preparation

HDPE/HDPE-*g*-MA/PA6 ternary blends were prepared by two step blend melting in a twin-screw extruder (TE-35, Jiangsu Nanjing Keya Co., Ltd., Nanjing, China). The HDPE-*g*-MA and PA6 were firstly blended to form HDPE-*g*-MA/PA6 binary blends in a twin-screw extruder, and then the resulting binary blends were blended with pure HDPE to form HDPE/HDPE-*g*-MA/PA6 ternary blends. The barrel temperature was set to 210, 220, 230, 230, 230, 240, and 240 °C from the hopper to the die in the extrusion step. Then all blends were injection molded using an injection molding machine (JPH-80, Dongguan step precision machine co., Ltd. Dongguan, China) at 240 °C to obtain standard specimens for the mechanical properties tests. Prior to melt mixing, all polymers were dried in an oven at 80 °C for 8 h before use to avoid moisture absorption. All ratios in the article correspond to weight ratio. For convenience studies, a large number of binary blends (HDPE/PA6, HDPE-*g*-MA/PA6) were also prepared.

### 2.3. Morphology Observation

The morphology of all the blends was characterized by scanning electron microscopy (SEM). The samples were fractured in two ways—low-temperature brittle fracture immersed in liquid nitrogen and impact fracture—and then covered with gold (sputtering method). Then the fractured samples were observed in a SEM instrument (S-4800, Hitachi, Tokyo, Japan) equipped with secondary electron detector at an acceleration voltage of 5 kV.

### 2.4. Polarized Optical Microscopy Observation

Polarized optical microscopy (POM) observations were carried out using an optical microscope (BX51, Olympus, Shenzhen, China) equipped with an Olympus camera and the temperature was monitored with a temperature controlled hot stage (THMS600, Linkam Co., Beijing, China). The specimens were sandwiched between two microscope cover slips for observation on the hot stage pressed with a cover slip to obtain a film of 80–100 µm thickness.

### 2.5. Contact Angle Measurements

Contact angles were measured in a sessile drop mold with a DSA100 (KRÜSS, Hamburg, German). HDPE, HDPE-*g*-MA, and PA6 samples were compression-molded between clean silicon wafers for 3 min and then cooled to 25 °C. under pressure for 1 min. The contact angle of each samples were measured using two different liquids (water, ethylene glycol).

### 2.6. Mechanical Tests

The blends were molded at 240 °C with a injection molding machine into dumb-bell shape samples. Tensile properties were measured at room temperature according to ASTM D-638 with an Instron tester at a crosshead speed of 50 mm/min. The Izod notched impact strength of the materials was measured with an XJUD-5.5 impact-testing machine (Chengde ZongChi testing instrument CO., LTD, Chengde, China). For each kind of blends, eight specimens were tested and the average value was given.

### 2.7. Dynamic Mechanical Analysis

The elasticity modulus of samples was measured using a DMS6100 dynamic thermomechanical analyzer (Hitachi, Tokyo, Japan) with alternating stress of the frequency of 1 Hz at a temperature rise rate of 2 °C/min. The specimen was prepared by cutting the standard sample with a volume of 35 × 10 × 2.5 mm^3^.

### 2.8. Differential Scanning Calorimetry Measurement

The crystallization behavior was examined by using a differential scanning calorimetry (DSC2500, TA Instruments, New castle, PA, USA) and the samples were placed in an aluminum pan. All operations were performed under a nitrogen atmosphere with a sample weight of about 5 mg. The DSC device is calibrated prior to use, including baseline calibration, temperature calibration, and energy calibration. Temperature and energy corrections were performed on the DSC equipment using the standard material—lead (Pb). The samples were first heated to 280 °C and held for 5 min to eliminate previous thermal history and remove impurities (impurities will be introduced during the experiment, such as water, which have a large effect on the experimental results), and then cooled down to 40 °C and maintained for 5 min. Finally, the samples were heated to 280 °C again. The cooling and twice-heating curves were recorded as the crystallization behavior. The heating and cooling rates in all tests were 10 and −5 °C/min, respectively.

## 3. Results and Discussion

### 3.1. The Phase Morphology

#### 3.1.1. The Phase Morphology Prediction

The phase morphology has an important effect on the properties of the materials, so it is necessary to predict the phase morphology of the ternary blends. The contact angles of different materials with water and ethynediol, and the surface tension, dispersion, and polar components of materials calculated by Equations (1) and (2) [32] are listed in Table 1.

(1) $$\left(1+\cos\theta_{H_{2}O}\right)\gamma_{H_{2}O}=4\left(\frac{\gamma_{H_{2}O}^{d}\gamma^{d}}{\gamma_{H_{2}O}^{d}+\gamma^{d}}+\frac{\gamma_{H_{2}O}^{p}\gamma^{p}}{\gamma_{H_{2}O}^{p}+\gamma^{p}}\right)$$

(2) $$\left(1+\cos\theta_{{\left({\mathrm{CH}}_{2}\mathrm{OH}\right)}_{2}}\right)\gamma_{{\left({\mathrm{CH}}_{2}\mathrm{OH}\right)}_{2}}=4\left(\frac{\gamma_{{\left({\mathrm{CH}}_{2}\mathrm{OH}\right)}_{2}}^{d}\gamma^{d}}{\gamma_{{\left({\mathrm{CH}}_{2}\mathrm{OH}\right)}_{2}}^{d}+\gamma^{d}}+\frac{\gamma_{{\left({\mathrm{CH}}_{2}\mathrm{OH}\right)}_{2}}^{p}\gamma^{p}}{\gamma_{{\left({\mathrm{CH}}_{2}\mathrm{OH}\right)}_{2}}^{p}+\gamma^{p}}\right)$$

(3) $$\gamma_{12}=\gamma_{1}+\gamma_{2}-4\left(\frac{\gamma_{1}^{d}\gamma_{2}^{d}}{\gamma_{1}^{d}+\gamma_{2}^{d}}+\frac{\gamma_{1}^{p}\gamma_{2}^{p}}{\gamma_{1}^{p}+\gamma_{2}^{p}}\right)$$

where γ, $\gamma^{d}$ and $\gamma^{p}$ are the surface tension, the dispersion component and the polar component, and θ is the contact angle with water or ethanediol. $\gamma_{12}$ is the interfacial tension between materials 1 and 2, $\gamma_{1}$ and $\gamma_{2}$ are the surface tensions of the two contacting components in the blends. The results about interfacial tensions calculated by Equation (3) are listed in Table 2. As can be seen from Table 1 and Table 2, the surface tension of HDPE-*g*-MA is 36, of which value is between HDPE and of PA6. The interfacial tension of HDPE/PA6 is 19, which is much greater than that of HDPE/HDPE-*g*-MA (4.4) and HDPE-*g*-MA/PA6 (8.8). As the phase morphology of a multicomponent polymer system is usual in the minimum free energy state, we can speculate that HDPE-*g*-MA will encapsulate PA6 forming shell-core structure particles dispersing in the matrix of HDPE according to the interface tension.

**Figure 1:**
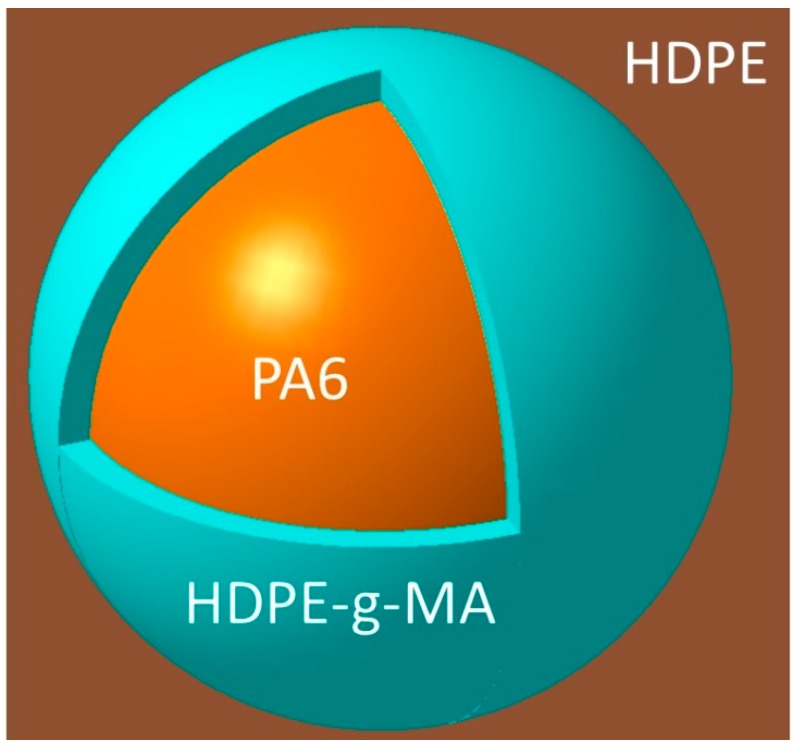
The schematic diagram of the core-shell structure.

Luzinov et al. [41] used Harkin’s equation to predict the phase morphology of ternary blends. For a ternary system with A as the major phase, and B and C as the dispersed phases, the spreading coefficients $\lambda_{\mathrm{BC}}$ and $\lambda_{\mathrm{CB}}$ are defined as:

(4) $$\lambda_{\mathrm{BC}}=\gamma_{\mathrm{AC}}-\gamma_{\mathrm{AB}}-\gamma_{\mathrm{BC}}$$

(5) $$\lambda_{\mathrm{CB}}=\gamma_{\mathrm{AB}}-\gamma_{\mathrm{AC}}-\gamma_{\mathrm{BC}}$$

where $\gamma_{\mathrm{AC}}$ is the interfacial tension between A and C phases. If the value of $\lambda_{\mathrm{BC}}$ is positive or the value of $\lambda_{\mathrm{CB}}$ is negative, the B phase will encapsulate the C phase forming the core-shell particles dispersed in the matrix of A. For the HDPE/HDPE-*g*-MA/PA6 ternary blends, using the above equation to calculate, $\lambda_{\mathrm{HDPE}-g-\mathrm{MA}/\mathrm{PA}6}$ and $\lambda_{\mathrm{PA}6/\mathrm{HDPE}-g-\mathrm{MA}}$ is 6 and −23.6, respectively, indicating that HDPE-*g*-MA will encapsulate PA6 forming shell-core structure particles dispersing in the matrix of HDPE. The schematic diagram of the core-shell structure is shown in Figure 1.

#### 3.1.2. The Phase Morphology Analysis

**Figure 2:**
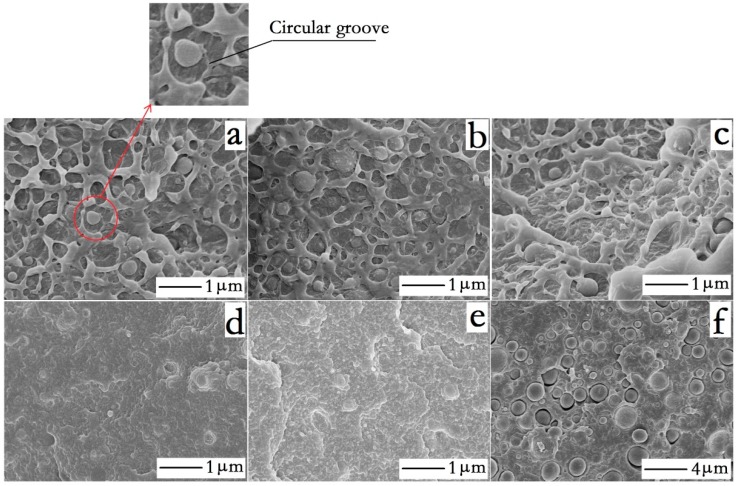
The fracture morphology of the HDPE/HDPE-*g*-MA/PA6 ternary system and several binary system: (**a**) HDPE/HDPE-*g*-MA/PA6 80/5/15, (**b**) HDPE/HDPE-*g*-MA/PA6 80/10/10 (**c**) HDPE/HDPE-*g*-MA/PA6 80/15/5, (**d**) HDPE/HDPE-*g*-MA 80/20, (**e**) HDPE-*g*-MA/PA6 25/75, and (**f**) HDPE/PA6 80/20.

Figure 2 shows the fracture morphology of the HDPE/HDPE-*g*-MA/PA6 ternary system and several (HDPE/PA6, HDPE-*g*-MA/PA6, HDPE/HDPE-*g*-MA) binary systems, of which samples are immersed in liquid nitrogen for 30 min before fracture.

From Figure 2d,e, the structure of the dispersed phase almost could not be observed for the HDPE-*g*-MA/PA6 and HDPE/HDPE-*g*-MA binary blends, showing a good compatibility between them. From the Figure 2f, a clear gap between the dispersed phase and the matrix is observed, indicating the poor compatibility between HDPE and PA6. However, the HDPE/HDPE-*g*-MA/PA6 ternary blends (Figure 2a–c) exhibit a very good adhesion between the dispersed phase and the matrix and the dispersed phase can be clearly seen in the matrix. Taking into account the above morphological prediction, we can speculate that HDPE-*g*-MA fills the gap between HDPE and PA6, forming a core-shell structure. Through comparing the morphology of ternary blends with the HDPE/PA6 binary blends, a wide circular groove could be observed around the dispersed phase of the ternary system (Figure 2a) while there is just a gap between the dispersed phase and the matrix for the HDPE/PA6 binary blends. The reason for this phenomenon is that HDPE-*g*-MA acting as the shell has different deformations compared to the other two materials when the sample fracture, leading to the formation of circular groove, which further confirms the existence of the core-shell structure.

For the ternary system, the dispersed phase surface is relatively rough. However, a smooth surface of the dispersed phase can be observed for the HDPE/PA6 binary system. This phenomenon may be attributed to the change of interface strength, of which reason is mainly as following. First of all, the addition of HDPE-*g*-MA has played a bridge role between HDPE and PA6, which reduces the interface energy and makes the polymer composite material have better compatibility. In addition, a very important reason is that the HDPE-*g*-MA will react with the amino end group of PA6, which will be discussed below, indicating the link between PA6 and HDPE-*g*-MA is not only the intermolecular force and the molecular chain entanglement, but also covalent bond. At the same time, the vinyl of HDPE-*g*-MA will insert into the HDPE matrix, which will undoubtedly improve the interface strength. When the interface strength is high (HDPE/HDPE-*g*-MA/PA6), the dispersion phase is more closely integrated with the matrix, and the interface will produce a large deformation at the time of fracture, resulting in the rough surface of the dispersed phase. For the HDPE/PA6 binary system, the interface strength is poor, and the surface of the dispersed phase does not produce a large deformation when the material fractures, so the dispersed phase exhibits a smooth surface. Since HDPE and PA6 are crystalline polymers, the crystallization process will lead to volume shrinkage [42]. Taking into account the poor adhesion between HDPE and PA6, HDPE and PA6 will undoubtedly produce gaps, breaking the material integrity.

As shown in Figure 2, the dispersed phase in the HDPE/PA6 binary system has a larger size (about 2 micrometer) compared with ternary system (about 0.2 micrometer). The reason is that PA6 is difficult to disperse into small particles in the melt blending process without the bridge role of HDPE-*g*-MA due to the high surface energy of PA6. Thus, the dispersed phase size of the HDPE/PA6 binary system is greater than the HDPE/HDPE-*g*-MA/PA6 ternary system.

In order to demonstrate the reaction between HDPE-*g*-MA and PA6, the HDPE-*g*-MA/PA6 binary blend with different ratios were dissolved in formic acid. When the content of PA6 is 75%, the samples are completely soluble in formic acid, forming a fine suspension which is still turbid after 24 h. This qualitative test—the so-called Molau test—demonstrates the presence of graft HDPE-*g*-MA/PA6 copolymers which acting as surfactants stabilizing a colloidal suspension of polyolefin particles in the PA6 solution [38]. When the content of PA6 is 50%, the sample cannot be dissolved by formic acid, but the volume is slightly swelling. The reason for this is that PA6 has almost completely reacted with HDPE-*g*-MA at this time, forming a large amount of graft copolymer, which is insoluble in formic acid due to the existence of a large amount of cross-linking between molecular chains. For comparative analysis, the HDPE/PA6 50/50 binary blend is also treated with formic acid, and the sample was found to be very fluffy, demonstrating that PA6 has been dissolved by formic acid.

**Figure 3:**
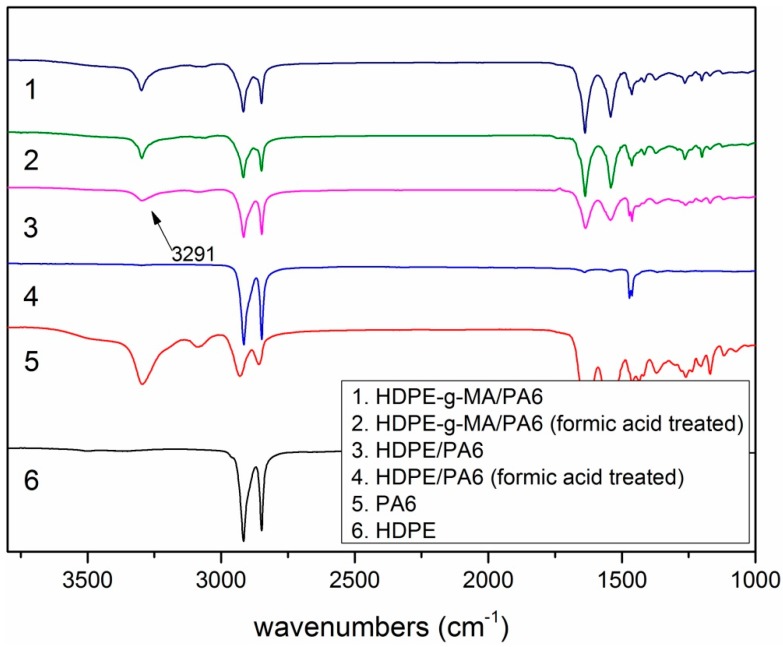
The FTIR spectra of HDPE, PA6, and the HDPE-*g*-MA/PA6 binary blend.

In order to further prove the above conjecture, the FTIR test (PE-983G, Perkin Elmer, Palm Springs, California, USA) was done. It can be seen from Figure 3 that the binary blends of HDPE-*g*-MA/PA6 and HDPE/PA6 have obvious absorption peaks at 3291 cm^−1^ (N–H stretching). But the absorption peak of HDPE/PA6 (50/50) at 3291 cm^−1^ disappears after the formic acid treatment, and the HDPE-*g*-MA/PA6 (50/50) has no change in the intensity of the absorption peak. These could prove that PA6 and HDPE-*g*-MA had completely reacted to form the graft copolymer and could not be dissolved in formic acid.

### 3.2. Crystallization Behavior Analysis

#### 3.2.1. Core-Shell Particle Crystallization Behavior

To explore the crystallization behavior of the core-shell (HDPE-*g*-MA/PA6) dispersed phase, several HDPE-*g*-MA/PA6 binary blends were prepared. Previously, many researchers have studied the crystallization behavior of HDPE-*g*-MA/PA6 binary blends. However, they often only studied a fixed composition ratio. This study investigated the crystallization behavior of individual components at different composition ratio.

**Figure 4:**
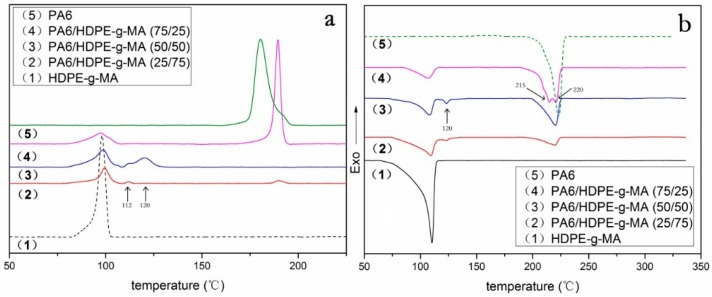
The DSC heating and cooling curve of HDPE-*g*-MA/PA6 blends. (**a**) cooling curve, and (**b**) heating curve.

To explore the crystallization process, Figure 4a shows the DSC cooling curve for the HDPE-*g*-MA/PA6 binary blend. As is shown in Figure 4a(1,5), the crystallization peaks of pure HDPE-*g*-MA and PA6 are near 98 and 180 °C, respectively. Figure 4a(2–4) are DSC cooling curves for the HDPE-*g*-MA/PA6 binary blend. With the increase of HDPE-*g*-MA content in the binary blend, the crystallization peak near 181 °C disappeared first and then appeared again. When the content of HDPE-*g*-MA was 50%, the crystallization peak near 181 °C completely disappeared and the new crystallization peaks appeared in the low-temperature region, which is consistent with the findings of previous researchers [37,43,44]. They consider that the PA6 droplets are so small that their number is probably much higher than that of the heterogeneous nuclei due to the addition of HDPE-*g*-MA. Therefore, a shift of the crystallization of PA6 to lower temperatures occurs. In fact, through the above experiments, we determined that this is not only a better dispersion, but that PA6 and HDPE-*g*-MA completely react to form a graft copolymer. The PA6 segment of the graft copolymer is affected by the HDPE segment and crystallizes at a lower temperature, so the double crystallized peaks are formed near 112 and 120 °C. When the content of HDPE-*g*-MA was 75%, although the PA6 was completely reacted at this time, a slight crystallization peak appeared near 190 °C. This may be because the HDPE-*g*-MA was excessive in the reaction and the polar group on HDPE-*g*-MA induces partial PA6 segments to crystallize at high temperatures, while the remaining PA6 segments still crystallize near 112 °C. Compared with the previous sample (50% HDPE-*g*-MA), the crystallization peak near 120 °C disappears. This may be because the content of the graft copolymer is less than the previous sample, and PA6 segments are less affected by the cross-linking molecular chains during crystallization, crystallizing only at 112 °C in the low-temperature region. When the content of HDPE-*g*-MA was 25%, a strong crystallization peak appeared on the DSC cooling curve near 190 °C. Because PA6 is excessive in this reaction, the unreacted PA6 is not well dispersed, leading to a high crystallization temperature. Since these unreacted PA6 crystallize first to occupy a large amount of crystallization space, the PA6 segment on the copolymer does not have sufficient crystallizing space. Therefore, there are no crystallization peaks at 112 and 120 °C.

To explore the type of crystallization, Figure 4b shows the heating DSC curve for the PA6/HDPE-*g*-MA binary blend. As shown in Figure 4b(5), pure PA6 has a single melting peak at 220 °C, indicating that pure PA6 forms stable α crystals. However, when the content of HDPE-*g*-MA was 25%, there were two melting peaks at 215 and 220 °C. The melting peak around 220 °C could be attributed to the melting of the α crystals. The one around 215 °C probably indicates the melting of the thermodynamically unstable γ crystals [45]. This is because the graft copolymer formed by the reaction of HDPE-*g*-MA with PA6 interferes with the crystallization of PA6, resulting in the formation of unstable γ crystals. When the content of HDPE-*g*-MA was 50% and 75%, the melting peak of 215 °C disappears, but a new melting peak appears at 120 °C. Combined with the previous analysis, we can speculate that partial γ crystals dissolve at 120 °C.

In order to study the crystallinity, crystallization temperature and melting temperature of binary blends, the summary of the DSC results are shown in Table 3.

The degree of crystallinity (*X*_C_) was calculated by Equation (6) [46]:

(6) $$X_{C}=\frac{\Delta H_{m}}{\Delta H_{m}^{0}\times W_{f}}$$

where $\Delta H_{m}$ is the measured melting enthalpy from DSC, $\Delta H_{m}^{0}$ is the enthalpy of the original polymer crystal (292 J/g for HDPE and 230 J/g for PA6) and $W_{f}$ is the weight fraction of each component in the blends. Here, we think that $\Delta H_{m\left(\mathrm{HDPE}-g-\mathrm{MA}\right)}^{0}$ is approximately the same as $\Delta H_{m\left(\mathrm{HDPE}\right)}^{0}$.

It can be seen from Table 3 that the crystallinity of PA6 first decreases and then increases with the increase of HDPE-*g*-MA content in the binary blend. When the content of HDPE-*g*-MA in the binary blend was 25% and 50%, the crystallinity of PA6 showed a slight decrease compared to pure PA6. The reason for this phenomenon is that the graft copolymer formed by reaction between PA6 and HDPE-*g*-MA interfered with the crystallization of PA6, resulting in a decrease in crystallinity. However, when the content of HDPE-*g*-MA is 75%, the crystallinity of PA6 rises sharply to 37%. There may be two reasons for this. One is that there is a large amount of unreacted HDPE-*g*-MA, of which the polar groups induce the crystallization of PA6. The other is that the crystallization temperature of PA6 is higher than HDPE-*g*-MA. When the HDPE-*g*-MA content in the blend is very high, PA6 has a large amount of crystallization space, resulting in an increase in crystallinity. While, the crystallinity of HDPE-*g*-MA always shows a decreasing trend with the increase of PA6 content in the binary blends. The reason for this phenomenon is that the increase of PA6 content will react with HDPE-*g*-MA to form more graft copolymers, which interfere with the crystallization of HDPE-*g*-MA. In addition, because the PA6 crystallizes before HDPE-*g*-MA in the binary blends, the increased PA6 will occupy more crystal space, which will lead to a decrease in the crystallinity of HDPE-*g*-MA.

When the content of HDPE-*g*-MA was 25%, the crystallization temperature of PA6 was 189.5 °C, which was greatly improved compared with pure PA6. These increases are clear evidence of nucleation by the polyethylene for the PA6 crystals, which is consistent with previous research [47]. When the content of HDPE-*g*-MA was 75% and 50%, the crystallization temperature of PA6 have been discussed above. The crystallization temperature of HDPE-*g*-MA increases first, and then decreases with the increase of PA6, but the change is not very large. The reason for this phenomenon is that PA6 can act as a nucleating agent, resulting in an increase in crystallization temperature when the content of PA6 is low in the binary blend. With the increase of PA6 content, the crystallization of PA6 which will occupy more crystal space interferes with the crystallization of HDPE-*g*-MA, resulting in a decrease in crystallization temperature.

The melting temperatures of both HDPE-*g*-MA and PA6 are reduced when the HDPE-*g*-MA and PA6 are mixed. The two polymers in the binary blend interfere with each other during crystallization process, resulting in forming more unstable crystal, such as the γ crystals of PA6, which will melt at lower temperatures.

#### 3.2.2. HDPE Matrix Crystallization Behavior

**Figure 5:**
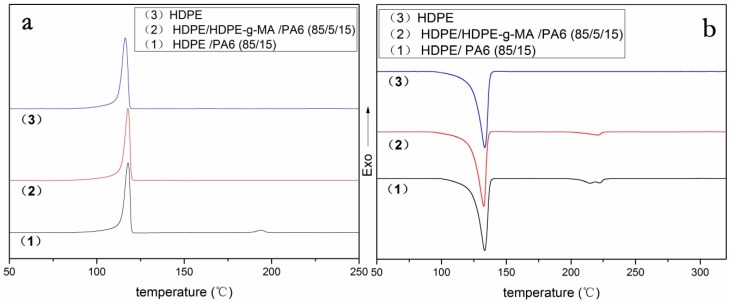
The DSC melting and cooling curve of PA6, HDPE-*g*-MA, HDPE, and their blends. (**a**) cooling curve, and (**b**) heating curve.

As shown in Figure 4a and Figure 5a, the binary blends HDPE/PA6 (85/15) and HDPE-*g*-MA/PA6 (25/75) have distinct crystallization peaks near 180 °C, while the HDPE/HDPE-*g*-MA/PA6 (80/5/15) ternary blend does not have this crystallization peak. Compared with the HDPE/PA6 (85/15) binary blend, the PA6 in the ternary blend is well dispersed due to the presence of HDPE-*g*-MA. Compared with the HDPE-*g*-MA/PA6 (25/75) binary blend, the PA6 in the ternary blend also has a better dispersion due to the larger dispersion space provided by the matrix of HDPE. Due to the good dispersion of PA6, the number of PA6 droplets is probably much higher than that of the heterogeneous nuclei, resulting in a decrease in crystallization temperature. Since the reduced crystallization temperature of PA6 was similar to that of HDPE, which has been discussed above, no significant PA6 crystallization peak was observed in Figure 5a(2).

As shown in Figure 5b(1), the binary blend HDPE/PA6 (85/15) have two PA6 melting peaks at 215 and 220 °C corresponding to α and γ crystal. However, the peak at 215 °C of the ternary blend disappeared (Figure 5b(2)). Due to the presence of HDPE-*g*-MA, a lot of the unstable γ crystal to dissolve at lower temperatures, just like the HDPE-*g*-MA/PA6 binary blend, leading to the disappearance of PA6 melting peaks at 215 °C. Similarly, since the lowered PA6 melting temperature is similar to that of HDPE, no significant melting peak is observed.

In order to study the crystallinity, crystallization temperature and melting temperature of ternary blend, the summary of the DSC results are shown in Table 4.

As shown in Table 4, the order of crystallinity of HDPE is HDPE < HDPE/PA6 < HDPE/HDPE-*g*-MA/PA6. Compared to pure HDPE, PA6 acts as a nucleating agent in the HDPE/PA6 binary blend, resulting in increased crystallinity. With the addition of HDPE-*g*-MA, PA6 is further dispersed, forming more cores, resulting in a greater crystallinity than the HDPE/PA6 binary blend. This can be further confirmed by the following study of grain size.

In the ternary blend, the crystallization temperature of HDPE is improved compared to pure HDPE, which may be due to the heterogeneous nucleation of PA6, leading to an increase in the crystallization ability of HDPE. In the ternary blend, the melting temperature of HDPE is lower than that of pure HDPE and binary blends. This phenomenon may be attributed to the body molecules produced by the reaction between HDPE-*g*-MA and PA6, which interfere with the crystallization of HDPE, resulting in the formation of many imperfect crystals, leading to a decrease in melting temperature.

**Figure 6:**
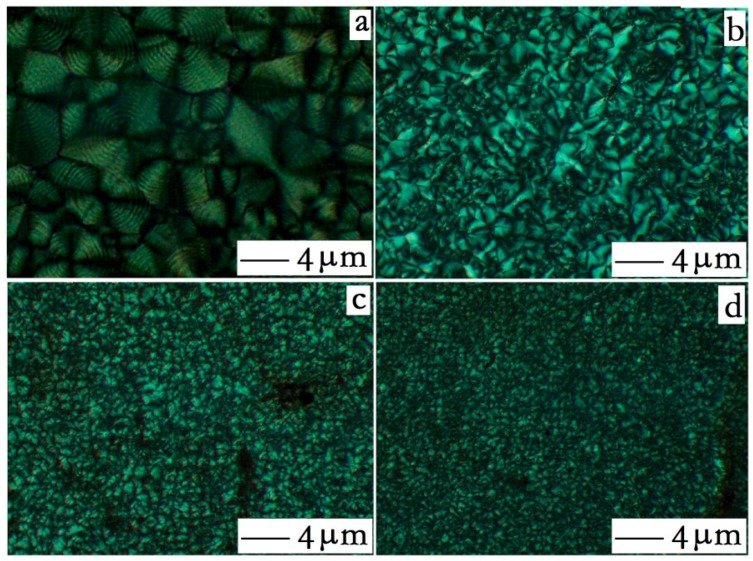
Polarized light microscopy. (**a**) HDPE, (**b**) PA6, (**c**) PA6/HDPE binary blends, and (**d**) HDPE/HDPE-*g*-MA/PA6 ternary blends.

In order to investigate the changes in grain size of ternary blends, polarized photographs of pure HDPE, pure PA6, HDPE/PA6 binary blends, and ternary blends are placed in Figure 6. As can be seen from the Figure 6a,b, both pure HDPE and pure PA6 have large grain sizes. While, the HDPE/PA6 binary blend (Figure 6c) has a grain size smaller than pure HDPE or PA6. The reason for this is that the PA6, as a nucleating agent, leads to a smaller HDPE grain size. The ternary blend (Figure 6d) has a grain size that is less than the binary blend. Due to the presence of HDPE-*g*-MA, the PA6 is sufficiently dispersed, which generated more nuclei, resulting in a smaller grain size.

### 3.3. Mechanical Performance Analysis

#### 3.3.1. Material Impact Properties

**Figure 7:**
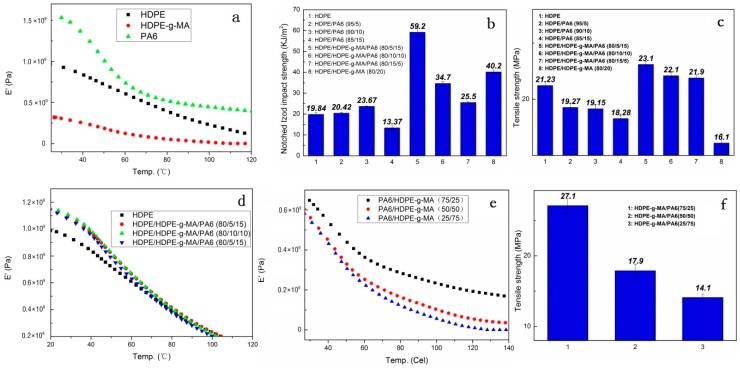
Mechanical properties of PA6, HDPE-*g*-MA, HDPE, and their blends. (**a**) the storage modulus of HDPE, HDPE-*g*-MA, and PA6, (**b**) the impact strength of blends, (**c**) the tensile strength of blends, (**d**) the storage modulus of ternary system, (**e**) the storage modulus of HDPE-*g*-MA/PA6 binary blends, and (**f**) the tensile strength of HDPE-*g*-MA/PA6 binary blends.

The storage modulus of HDPE, HDPE-*g*-MA, and PA6 determined by DMA is shown in Figure 7a. The storage modulus of the material can be used to represent the elastic modulus. It can be seen that the HDPE-*g*-MA is considered to be a rubber phase because of its low modulus of elasticity, the modulus of PA6 is the highest, and the modulus of HDPE is between the two.

The impact strength of the HDPE/HDPE-*g*-MA/PA6 ternary system is shown in the Figure 7b. It can be seen that the addition of core-shell particles (HDPE-*g*-MA/PA6) does increase the impact strength of HDPE (maximum value 59 kJ/m^2^), which is close to three times than the pure HDPE. This is consistent with the conclusion that some of the previous researchers have concluded that the core-shell structure can improve the impact strength [31,32,33]. The toughening mechanism has also been discussed by many people [33,34,35]. Chen et al. [34] propose that the addition of the core decreases the distance between the dispersed phases, so the rubber phase as a shell in the core-shell particles can more effectively induce crazing and shear yielding of the matrix to absorb the breaking energy, resulting in an increase in impact strength. Through the study of HDPE/HDPE-*g*-MA binary system, it is found that this is very reasonable. The binary system HDPE/HDPE-*g*-MA (80/20) has an impact strength of 40 kJ/m^2^, which is twice that of pure HDPE. This shows that HDPE-*g*-MA does have a toughening effect on HDPE, which can be explained by the mechanism of elastomer toughening. In addition, the impact strength of HDPE/HDPE-*g*-MA (80/20) is greater than that of HDPE/HDPE-*g*-MA/PA6 (80/10/10 or 80/15/5), which may be attributed to the smaller distance between the dispersed phases. As is shown in the Figure 2d, the dispersed phase morphology could not be observed, which means that their compatibility is very good, and the distance between the dispersed phases must be less than the HDPE/HDPE-*g*-MA/PA6 (80/10/10 or 80/15/5). This is consistent with Chen’s research. However, as can be seen from Figure 2a, the dispersion phase distance of the ternary blend HDPE/HDPE-*g*-MA/PA6(80/5/15) is necessarily greater than that of HDPE/HDPE-*g*-MA (80/20) binary blend, but its impact strength is better than that of binary blend. In addition, there is no difference in the dispersion phase distance of the three component ternary blends (Figure 2a–c), but their impact strength is quite different, which is inconsistent with Chen’s theory. All of this shows that there must be other ways to absorb the fracture energy, resulting in changes in impact strength. Shen et al. [33] proposed that the addition of a rigid core can prevent the rupture of the rubber phase, resulting in an increase in impact strength. Yin et al. [35] proposed that the good stress transfer effect between dispersed particles and polymer matrix plays an important role in toughening the polymer. In fact, these two statements are essentially the same. They all mean that the core-shell particles absorb a large amount of fracture energy when they are stretched, resulting in an increase in impact strength. Combined with this factor, the above phenomenon can be explained. Although the distance between the dispersed phases of the HDPE/HDPE-*g*-MA/PA6 (80/5/15) ternary system is very large, the core-shell particle in the ternary system own high strength and elastic modulus due to the presence of PA6, which will absorb a large amount of fracture energy under stretching, resulting in a greater impact of the ternary system than the binary HDPE/HDPE-*g*-MA (80/20).

**Figure 8:**
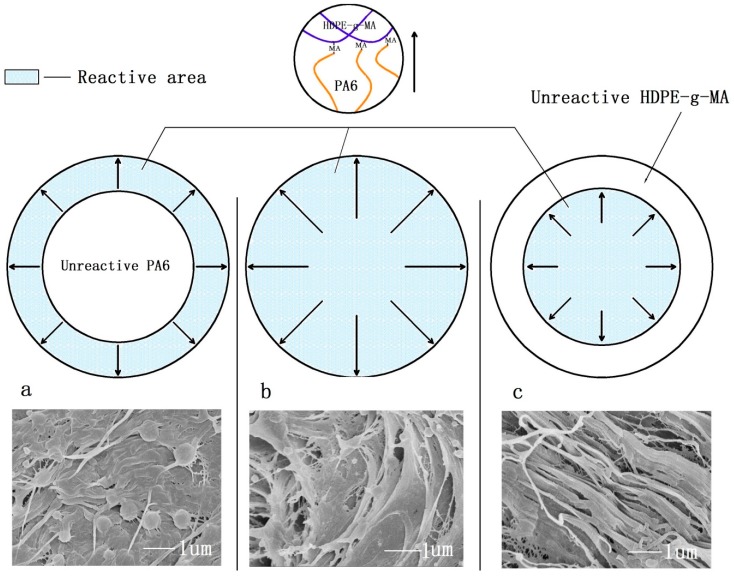
(**a**) HDPE/HDPE-*g*-MA/PA6 (80/5/15), (**b**) HDPE/HDPE-*g*-MA/PA6 (80/10/10), and (**c**) HDPE/HDPE-*g*-MA/PA6 (80/15/5).

Figure 8 illustrates why the impact strength of the samples are different when the ratio of the core to shell was changed. The tensile strength and storage modulus of HDPE-*g*-MA/PA6 binary blends decrease with the increase of HDPE-*g*-MA content, which is shown in the Figure 7e–f. For the HDPE/HDPE-*g*-MA/PA6 (80/5/15) ternary system, the HDPE-*g*-MA will react fully with PA6, of which the schematic diagram is displayed in Figure 8a. The elastic modulus and interface strength of the whole core-shell particles is very high due to the natural high modulus of PA6 and the covalent bonds between PA6 and HDPE-*g*-MA. It can be seen from the scanning electron micrograph that the dispersed phase maintains a spherical morphology after being impacted due to the presence of unreacted PA6 inside. At this time, the strength of the dispersed phase is high, which will absorb a large amount of energy during the stretching process, resulting in increased impact strength. However, with the proportion of HDPE-*g*-MA increases, the tensile strength and elastic modulus of the whole core-shell particles will decreases, which will absorbs less energy than before. Therefore, the impact strength of the ternary system decreases with the increases of HDPE-*g*-MA content. For the HDPE/HDPE-*g*-MA/PA6 (80/10/10) ternary system, no obvious dispersed phase was observed on the SEM image. Since the HDPE-*g*-MA is almost completely reacted with PA6 in this composition, the dispersed phase is stretched as a whole (Figure 8b). For the HDPE/HDPE-*g*-MA/PA6 (80/15/5) ternary system, the HDPE-*g*-MA is excessive in the reaction between PA6 and HDPE-*g*-MA. The unreacted HDPE-*g*-MA must be distributed on the outer side of the core-shell particles leading to a very low interfacial strength between the shell-core particles and matrix, of which the schematic diagram is displayed in Figure 8c. As the elasticity modulus of the unreacted HDPE-*g*-MA is very low, it will deformed rapidly when the core-shell particles are stretched to form a fibrous structure. Although producing a large deformation, the absorption of fracture energy is not very much due to the low modulus of HDPE-*g*-MA. At the same time, there are no covalent bonds between the unreacted HDPE-*g*-MA and PA6, and the tensile force will not effectively be transferred to inner PA6, impeding the absorption of fracture energy. All of these lead to the decrease in impact strength.

For the HDPE/PA6 binary system, which does not form a core-shell structure, the impact strength is lower than that of HDPE/HDPE-*g*-MA/PA6 ternary system. The HDPE/PA6 binary system is equivalent to the introduction of rigid particles into the HDPE matrix. When the PA6 content is less, the impact strength will have a slight degree of improvement due to the toughening mechanism of rigid particles. However, because of the poor interface strength, the amount of defects in the binary blends will increase with the increase of PA6 content, resulting in a sharp drop in impact strength.

**Figure 9:**
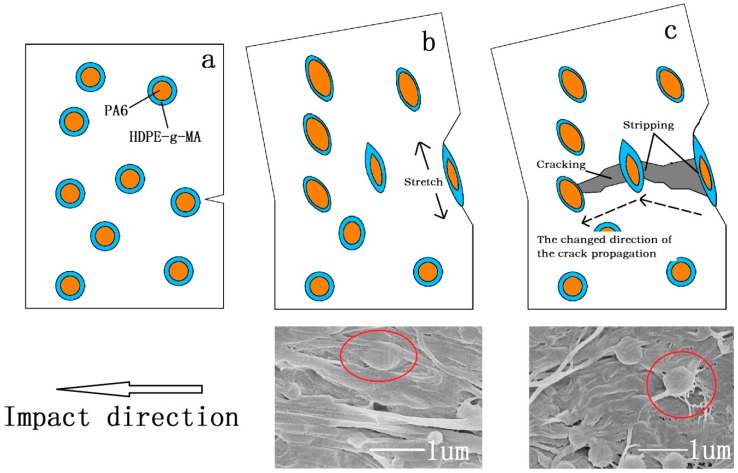
Toughening mechanism of the core-shell structure. (**a**) undeformed core-shell particles, (**b**) stretched core-shell particles, and (**c**) the stripping of core-shell particles and the change of cracking propagation direction.

Through the above study, the mechanism of the core-shell structure toughening is not only one, but the result of a variety of mechanisms together. In the process of sample fracture, the energy consumption path is shown in the Figure 9. First, as the stress concentration on the low-modulus shell, the core-shell particles will induce crazing and shear yielding to absorb energy, which is the same as with the toughening mechanism of the elastomer. At the same time, the addition of rigid core (PA6) decreasing the distance among dispersion phase can more efficient absorb energy (Figure 9a). Second, the core-shell particles will be stretched from the original circular particles into reinforced fibers when the sample is deformed upon stress. Compared with the use of low strength and modulus materials as the core, PA6 own high elastic modulus and strength, which will absorb more energy. The SEM image of the impact fracture (Figure 9b) shows the stretched traces of the core-shell particles. Third, when the core-shell particles are stretched, the core-shell particles will be stripped from the matrix due to the cracking propagation, which must absorb much energy due to the high interfacial strength. From the SEM photograph of impact fracture (Figure 9c), the stripping traces of stretched core-shell particles can be seen. Finally, because of the presence of core-shell particles, the direction of the crack propagation must be changed, producing a rough fracture surface, which must absorb a great deal of fracture energy.

#### 3.3.2. Tensile Strength and Elastic Modulus

It can be seen from the Figure 7c that the tensile strength of the HDPE/HDPE-*g*-MA/PA6 ternary system is higher than that of pure HDPE, while the tensile strength of the HDPE/PA6 binary system is lower than that of pure HDPE. For the HDPE/HDPE-*g*-MA/PA6 (80/5/15) ternary system, the addition of HDPE-*g*-MA improves the interfacial strength between HDPE and PA6, which could effectively transfer the load from HDPE matrix to PA6 particles. The strength of the core-shell particles is higher than that of the HDPE matrix, playing a reinforced role here. Moreover, the crystallinity of HDPE is enhanced by the addition of core-shell particles, which will also enhance the tensile strength. For the HDPE/HDPE-*g*-MA/PA6 (80/10/10 or 80/15/5) ternary system, the strength of the dispersed phase is smaller than that of the matrix. Therefore, its enhancement may be due only to the increase in crystallinity of the HDPE matrix. In HDPE/PA6 binary system, a large number of defects were produced around the PA6 particles due to the poor interface strength between HDPE and PA6, resulting in the decreased tensile strength.

The effect of the core-shell structure on the elastic modulus of HDPE is shown in the Figure 7d. At room temperature, the addition of core-shell particles greatly increases the storage modulus of the material, about 1.2 times. Since the good interfacial strength between the core-shell particles and the matrix, and the crosslinking between molecular chains, the elastic modulus of the polymer blends must be high. When the temperature rises to 60 °C, the storage modulus of HDPE/HDPE-*g*-MA/PA6 ternary system becomes almost the same as that of HDPE. The reason is that PA6 undergoes the glass transition at that temperature and the elastic modulus decrease.

In this paper, HDPE was modified by the HDPE-*g*-MA/PA6 core-shell particles. Different from previous research, the HDPE-*g*-MA as a shell can not only react with PA6, but also has a good compatibility with the HDPE matrix due to the presence of vinyl, resulting in a high interface strength in both sides of the shell. In addition, the strength and modulus of the core (PA6) is higher than that of the HDPE matrix, so that the ternary blend will have an increase in strength and modulus compared to pure HDPE. All of these will improve the comprehensive mechanical properties of HDPE, such as tensile strength, elastic modulus, and impact strength, avoiding the shortcomings of increasing the toughness leading to a loss of strength and modulus in the previous research.

## 4. Conclusions

In this paper, HDPE was modified by the HDPE-*g*-MA/PA6 core-shell particles. Through theoretical calculation, infrared spectroscopy, and SEM morphology analysis, it was determined that the ternary blend formed a core-shell structure. The crystallization behavior of each component in the ternary blend was analyzed by DSC testing. The crystallization behavior of the HDPE-*g*-MA/PA6 binary blends which act as the core-shell particles in this work have been studied in detail. The reaction between HDPE-*g*-MA and PA6 will have a significant effect on the crystallization of blends, such as crystallization process, crystallinity, crystallization temperature and melting temperature. The effect of core-shell particles on the crystallization behavior of HDPE matrix has also been studied. Through the mechanical properties testing, the comprehensive mechanical properties of ternary blends have been greatly improved, such as tensile strength, elastic modulus and impact strength. The HDPE/HDPE-*g*-MA/PA6 (80/5/15) ternary blends reflected the best performance compared to other proportions, especially the impact strength, which is close to three times than the pure HDPE. The toughening mechanism of core-shell structure is not a single, but a variety of synergistic results. The effect of different core-shell ratios on mechanical properties has also been discussed in detail.

In my opinion, the use of fiber-like reinforcements in combination with core-shell particles to modify polymers is a good direction for future work.

## Figures and Tables

**Table 1:** Summary of contact angle and surface tension of the blends.

Sample	Contact	Angle (°)	Surface Tension		(mN/m)
	Water	Ethanediol	Total (γ)	Dispersion Component (γ^d^)	Polar Component (γ^p^)
HDPE	95.7	69.9	24.3	16	8.3
HDPE-*g*-MA	73.4	46.1	36.1	17.4	18.7
PA6	64.2	60.5	41.7	7.2	34.5

**Table 2:** Interfacial tension of each blend (calculated by Equation (3)).

Polymer Pairs	Interfacial Tension (mN/m)
HDPE/ HDPE-*g*-MA	4.4
HDPE/PA6	19.2
HDPE-*g*-MA/PA6	8.8

**Table 3:** Melting temperature (*T*_m_), crystallization temperature (*T*_c_), melting enthalpy ($\Delta H_{m}$), and the degree of crystallinity (*X*_c_) for HDPE-*g*-MA/PA6 blends.

PA6/HDPE-*g*-MA (*w*/*w*)	$\Delta H_{m}$ (J/g)		*T*_m_ (°C)		*T*_c_ (°C)		*X*_c_ (%)	
	PA6	HDPE-*g*-MA	PA6	HDPE-*g*-MA	PA6	HDPE-*g*-MA	PA6	HDPE-*g*-MA
0/100	-	120	-	110.2	-	98.1	-	41
25/75	21.2	59.2	123.5/220.3	109.6	112.1/189.7	99.1	37	27
50/50	33.3	37.8	123.5/220.1	108.0	111.8/120.4	98.5	29	26
75/25	50.2	15.0	215.1/220.7	107.3	189.5	97.6	29	21
100/0	69.1	-	223	-	180.4	-	30	-

**Table 4:** Melting temperature (*T*_m_), crystallization temperature (*T*_c_), melting enthalpy ($\Delta H_{m}$), and the degree of crystallinity (*X*_c_) for HDPE, HDPE/PA6, and HDPE/HDPE-*g*-MA/PA6 blends.

HDPE/HDPE-*g*-MA/PA6 (*w*/*w*/*w*)	$\Delta H_{m}$ (J/g)		*T*_m_ (HDPE) (°C)		*T*_c_ (HDPE) (°C)		*X*_c_ (HDPE) (%)	
	HDPE	PA6	HDPE	PA6	HDPE	PA6	HDPE	PA6
85/0/15	153	9.3	133.3	215/220	117.8	194	62	30
80/5/15	153	-	132.7	-	117.9	-	65	-
100/0/0	179	-	133.3	-	116.2	-	61	-

## References

[1] Bajracharya R.M., Manalo A.C., Karunasena W., Lau K.T. (2016). Experimental and theoretical studies on the properties of injection molded glass fiber reinforced mixed plastics composites. Compos. Part A.

[2] Lu X., Tang L., Wang L., Zhao J., Li D., Wu Z., Xiao P. (2016). Morphology and properties of bio-based poly(lactic acid)/high-density polyethylene blends and their glass fiber reinforced composites. Polym. Test..

[3] Perez C.J., Alvarez V.A. (2016). Ternary composites based on hdpe and Mater-Bi reinforced with hemp fibers. J. Therm. Anal. Calorim..

[4] Kuzmanović M., Delva L., Mi D., Martins C., Cardon L., Ragaert K. (2018). Development of crystalline morphology and its relationship with mechanical properties of PP/PET microfibrillar composites containing POE and POE-*g*-MA. Polymers.

[5] Zhang P., Wang B. (2018). Positive temperature coefficient effect and mechanism of compatible LLDPE/HDPE composites doping conductive graphite powders. J. Appl. Polym. Sci..

[6] Zhang Q., Cai H., Ren X., Kong L., Liu J., Jiang X. (2017). The Dynamic Mechanical Analysis of Highly Filled Rice Husk Biochar/High-Density Polyethylene Composites. Polymers.

[7] Srivastava P., Sinha S. (2018). Effect of surface treatment on hair fiber as reinforcement of HDPE composites: Mechanical properties and water absorption kinetics. Korean J. Chem. Eng..

[8] Zhang L., Lv S., Sun C., Wan L., Tan H., Zhang Y. (2017). Effect of mah-*g*-pla on the properties of wood fiber/polylactic acid composites. Polymers.

[9] Lee S., Kim M.S., Ogale A.A. (2010). Influence of carbon nanofiber structure on properties of linear low density polyethylene composites. Polym. Eng. Sci..

[10] Jang S.H., Kim Y.H., Lim S., Choi G.D., Kim S.H., Kim W.N. (2010). Effects of fiber characteristics on the mechanical and rheological properties of poly(butylene terephthalate)/glass fiber composites. J. Appl. Polym. Sci..

[11] Lv Y., Peng C. (2018). Chemically grafting carbon nanotubes onto carbon fibers for enhancing interfacial strength in carbon fiber/HDPE composites. Surf. Interface Anal..

[12] Zhang S., Ke Y., Cao X., Ma Y., Wang F. (2012). Effect of Al_2_O_3_ fibers on the thermal conductivity and mechanical properties of high density polyethylene with the absence and presence of compatibilizer. J. Appl. Polym. Sci..

[13] Liu W., Wang L., Mai J., Zhang A. (2014). Synthesis of EPDM-*g*-MAN by suspension graft copolymerization and its toughening effect on SAN resin. Polym. Bull..

[14] Mao Z., Zhang J. (2018). Largely improved the low temperature toughness of acrylonitrile-styrene-acrylate (ASA) resin: Fabricated a core-shell structure of two elastomers through the differences of interfacial tensions. Appl. Surf. Sci..

[15] Jung H. (2015). Carbon fiber-reinforced plastics based on epoxy resin toughened with core shell rubber impact modifiers. Polymers..

[16] Sun D.X., Yang C.J., Qi X.D., Yang J.H., Wang Y. (2018). Largely enhanced fracture toughness of the PP/EPDM blends induced by adding carbon nanofibers. Compos. Sci. Technol..

[17] Mansour G., Tsongas K., Tzetzis D. (2016). Investigation of the dynamic mechanical properties of epoxy resins modified with elastomers. Compos. Part B.

[18] Chi X., Cheng L., Liu W., Zhang X., Li S. (2018). Characterization of Polypropylene Modified by Blending Elastomer and Nano-Silica. Materials.

[19] Douse E., Kopsidas S., Jesson D., Hamerton I. (2016). Modification of stress-strain behavior in aromatic polybenzoxazines using core shell rubbers. React. Funct. Polym..

[20] Yuan Q., Caillard J., Rong L. (2018). Fracture toughness of soft materials with rate-independent hysteresis. J. Mech. Phys. Solids.

[21] Wal A.V.D., Mulder J.J., Oderkerk J., Gaymans R.J. (1998). Polypropylene-rubber blends: 1. the effect of the matrix properties on the impact behavior. Polymer.

[22] Badamshina E.R., Goldstein R.V., Ustinov K.B., Estrin Y.I. (2018). Strength and fracture toughness of polyurethane elastomers modified with carbon nanotubes. Phys. Mesotech..

[23] Wal A.V.D., Nijhof R., Gaymans R.J. (1999). Polypropylene-rubber blends: 2. the effect of the rubber content on the deformation and impact behavior. Polymer..

[24] Chaudhary S., Surekha P., Kumar D., Rajagopal C., Roy P.K. (2015). Amine-functionalized poly(styrene) microspheres as thermoplastic toughened for epoxy resin. Polym. Compos..

[25] Keivani M., Khamesinia A., Bagheri R., Kouchakzadeh M.A., Abadyan M. (2015). Study of synergistic toughening in a bimodal epoxy nanocomposite. J. Reinf. Plast. Compos..

[26] Kurauchi T., Ohta T. (1984). Energy absorption in blends of polycarbonate with abs and san. J. Mater. Sci..

[27] Sprenger S. (2013). Epoxy resins modified with elastomers and surface-modified silica nanoparticles. Polymer.

[28] Bucknall C.B., Paul D.R. (2013). Notched impact behavior of polymer blends: Part 2: Dependence of critical particle size on rubber particle volume fraction. Polymer.

[29] Zuiderduin W.C.J., Westzaan C., Huétink J., Gaymans R.J. (2003). Toughening of polypropylene with calcium carbonate particles. Polymer.

[30] Argon A.S., Cohen R.E. (2003). Toughen ability of polymers. Polymer.

[31] Wang B., Wu D., Zhu L., Jin Z., Zhao K. (2017). High-density polyethylene-based ternary blends toughened by PA6/PBT core-shell particles. Polym. Plast. Technol..

[32] Li L.P., Yin B., Zhou Y., Gong L., Yang M.B., Xie B.H. (2012). Characterization of PA6/EPDM-*g*-MA/HDPE ternary blends: The role of core-shell structure. Polymer.

[33] Shen C., Zhou Y., Dou R., Wang W., Yin B., Yang M.B. (2015). Effect of the core-forming polymer on phase morphology and mechanical properties of PA6/EPDM-*g*-MA/HDPE ternary blends. Polymer.

[34] Chen F., Shangguan Y., Jiang Y., Qiu B., Luo G., Zheng Q. (2015). Toughening with little rigidity loss and mechanism for modified polypropylene by polymer particles with core-shell structure. Polymer.

[35] Yin B., Li L.P., Zhou Y., Gong L., Yang M.B., Xie B.H. (2013). Largely improved impact toughness of PA6/EPDM-*g*-MA/HDPE ternary blends: The role of core-shell particles formed in melt processing on preventing micro-crack propagation. Polymer.

[36] Valera T.S., And A.T.M., Demarquette N.R. (2006). Study of morphologies of PMMA/PP/PS ternary blends. Macromolecules.

[37] Filippi S., Chiono V., Polacco G., Paci M., Minkova L.I., Magagnini P. (2002). Reactive compatibilizer precursors for LDPE/PA6 blends, 1. Ethylene/acrylic acid copolymers. Macromol. Chem. Phys..

[38] Jiang C., Filippi S., Magagnini P. (2003). Reactive compatibilizer precursors for LDPE/PA6 blends. ii: Maleic anhydride grafted polyethylenes. Polymer.

[39] Kord B., Roohani M. (2017). Water transport kinetics and thickness swelling behavior of natural fiber-reinforced hdpe/cnt nanocomposites. Compos. Part B.

[40] Bian J., Wang G., Lin H.L., Zhou X., Wang Z.J., Xiao W.Q. (2017). HDPE composites strengthened-toughened synergistically by l-aspartic acid functionalized graphene/carbon nanotubes hybrid nanomaterials. J. Appl. Polym. Sci..

[41] Luzinov I., Xi K., Pagnoulle C., Huynh-Ba G., Jérôme R. (1999). Composition effect on the core-shell morphology and mechanical properties of ternary polystyrene/styrene-butadiene rubber/polyethylene blends. Polymer.

[42] Shangguan Y., Chen F., Yang J., Jia E., Zheng Q. (2017). A new approach to fabricate polypropylene alloy with excellent low-temperature toughness and balanced toughness-rigidity through unmatched thermal expansion coefficients between components. Polymer.

[43] Ohlsson B., Hassander H., Törnell B. (1998). Effect of the mixing procedure on the morphology and properties of compatibilized polypropylene/polyamide blends. Polymer.

[44] Psarski M., Pracella M., Galeski A. (2000). Crystal phase and crystallinity of polyamide 6/functionalized polyolefin blends. Polymer.

[45] Klata E., Velde K.V.D., Krucińska I. (2003). Dsc investigations of polyamide 6 in hybrid GF/PA6 yarns and composites. Polym. Test..

[46] Åkesson D., Fuchs T., Stöss M., Root A., Stenvall E., Skrifvars M. (2016). Recycling of wood fiber-reinforced HDPE by multiple reprocessing. J. Appl. Polym. Sci..

[47] Charoenpongpool S., Nithitanakul M., Grady B.P. (2013). Melt-neutralization of maleic anhydride grafted on high-density polyethylene compatibilizer for polyamide-6/high-density polyethylene blend: Effect of neutralization level on compatibility of the blend. Polym. Bull..

